# BipD of *Burkholderia pseudomallei*: Structure, Functions, and Detection Methods

**DOI:** 10.3390/microorganisms9040711

**Published:** 2021-03-30

**Authors:** Kasturi Selvam, Muhammad Fazli Khalid, Khairul Mohd Fadzli Mustaffa, Azian Harun, Ismail Aziah

**Affiliations:** 1 Institute for Research in Molecular Medicine (INFORMM), Health Campus, Universiti Sains Malaysia, Kubang Kerian 16150, Kelantan, Malaysia; kasturiselvam0612@gmail.com (K.S.); fazlikhalid@usm.my (M.F.K.); khairulmf@usm.my (K.M.F.M.); 2 Department of Medical Microbiology and Parasitology, School of Medical Sciences, Health Campus, Universiti Sains Malaysia, Kubang Kerian 16150, Kelantan, Malaysia; azian@usm.my

**Keywords:** melioidosis, *B. pseudomallei*, BipD, aptasensor

## Abstract

Melioidosis is a severe disease caused by *Burkholderia pseudomallei* (*B. pseudomallei*), a Gram-negative environmental bacterium. It is endemic in Southeast Asia and Northern Australia, but it is underreported in many other countries. The principal routes of entry for *B. pseudomallei* are skin penetration, inhalation, and ingestion. It mainly affects immunocompromised populations, especially patients with type 2 diabetes mellitus. The laboratory diagnosis of melioidosis is challenging due to its non-specific clinical manifestations, which mimic other severe infections. The culture method is considered an imperfect gold standard for the diagnosis of melioidosis due to its low sensitivity. Antibody detection has low sensitivity and specificity due to the high seropositivity among healthy people in endemic regions. Antigen detection using various proteins has been tested for the rapid determination of *B. pseudomallei*; however, it presents certain limitations in terms of its sensitivity and specificity. Therefore, this review aims to frame the present knowledge of a potential target known as the *Burkholderia* invasion protein D (BipD), including future directions for its detection using an aptamer-based sensor (aptasensor).

## 1. Introduction

*B. pseudomallei* is a motile, Gram-negative environmental bacterium. It is the etiological agent of melioidosis, also known as Whitmore’s disease, which affects humans and a wide range of animals such as hamsters, sheep, monkeys, and dolphins [1]. Melioidosis is highly endemic in Northern Australia and Southeast Asia, particularly in Northeast Thailand [2]. Cases are usually imported from these areas into developed countries such as those in Europe and the United States [3,4]. The case reports of melioidosis and predictive modeling studies indicate that it is present in many countries but is underreported [5]. In Malaysia, most melioidosis case reports are from hospitals and medical centers in Pahang and Sabah [6]. Globally, *B. pseudomallei* causes an estimated 165,000 cases of human melioidosis and 89,000 deaths each year [5]. Furthermore, it is responsible for 20% of all community-acquired septicemias and 40% of the mortality due to sepsis in northern Thailand [7].

*B. pseudomallei* is a natural saprophyte and is commonly discovered in soil and muddy water throughout endemic areas [8]. It is mainly transmitted to humans through the inoculation of the skin, inhalation, or ingestion [9]. Melioidosis is not contagious; however, human-to-human transmission has been reported [10]. The zoonotic transmission of melioidosis to humans from contact with livestock is sporadic [11]. Melioidosis can present with various clinical signs and symptoms; thus, *B. pseudomallei* is known as the great mimicker [12]. The clinical presentations typically range from the development of abscesses in the skin and soft tissue to acute pneumonia and fatal septicemia [13]. Additionally, the power of *B. pseudomallei* to infect almost every tissue type leads to the development of various other manifestations such as encephalitis, osteomyelitis, and abscesses in internal organs [14].

*B. pseudomallei* often affects adults with one or more underlying predisposing conditions or compromised immune responses. Patients with diabetes mellitus, chronic kidney disease, binge alcohol consumption, and cystic fibrosis are grouped as being at high risk of melioidosis [15,16]. The most common risk factor is diabetes mellitus (DM), usually type 2, as 40–60% of melioidosis patients have diagnosed type 2 DM [14]. The typically reported incubation period for melioidosis is 1–21 days after infection [10]. *B. pseudomallei* is reactivated after latent infection and contributes to an acute fatal disease, mainly when the immune system is suppressed. The longest latency period was reported from a World War II veteran who exhibited symptoms of melioidosis 62 years after a primary exposure [17].

*B. pseudomallei* is resistant to the majority of antibiotics; thus, treatment choices are severely limited [18]. High-dose parenteral therapy for at least 10 days is recommended in systemic infections. Then, oral eradication therapy with high-dose trimethoprim–sulfamethoxazole is used to complete the treatment for a full 20 weeks [19]. Even though effective antibiotics are available, the treatment for melioidosis requires prolonged therapy to prevent relapses [20]. Melioidosis leads to death in 10% of infected individuals, even if properly treated; the mortality rate can rise to 40% with poor treatment [8]. *B. pseudomallei* was listed as a category B bio-threat agent by the Centre for Disease Control and Prevention in 2002 due to its potential to cause life-threatening airborne infections and its limited therapeutic alternatives. At present, there is no available vaccine against *B. pseudomallei* [21].

The laboratory identification of *B. pseudomallei* is challenging due to its non-specific clinical signs and symptoms that commonly resemble tuberculosis. The culture of *B. pseudomallei* is an imperfect gold standard for melioidosis diagnosis due to its low sensitivity (70.3%) [22]. *B. pseudomallei* is frequently misidentified as a *Pseudomonas* species because of similar colony morphology in blood agar, Gram staining (Gram-negative and safety-pin appearance), and biochemical tests such as the oxidase test (oxidase positive) [23]. The detection of *B. pseudomallei* is difficult in routine culture media because it mimics contaminants, and the overgrowth of normal flora is observed [22]. Furthermore, the culture method is time-consuming, as it takes five to seven days and requires selective or enriched media for non-sterile samples. It also requires a class III biosafety cabinet, and experts are often unavailable in endemic areas [24,25].

The indirect hemagglutination assay (IHA) has been widely used to determine antibody titers and can be used as an indicator of *B. pseudomallei* exposure [26]. However, the presence of high background antibodies due to previous exposure to *B. pseudomallei* and closely related environmental species, especially *B. thailandensis*, leads to low specificity and sensitivity as well as the inability to monitor treatment responses [27]. Other potential problems with IHA are the various and unstandardized strains used for antigen preparation in different laboratories and a short shelf-life [28].

The immunofluorescent assay (IFA) and latex agglutination (LA) provide rapid confirmation of a melioidosis diagnosis, in which a monoclonal antibody (mAb 4B11) recognizes the capsular polysaccharide (CPS) of *B. pseudomallei* in positive blood cultures [29,30]. However, the marked disadvantages of IFA are a tendency to misidentify fluorescent debris as bacteria, and the labor- and resource-intensive methodology [25]. Furthermore, LA reagents are not commercially available and require cold-chain storage [30].

With regards to molecular methods, many types of polymerase chain reactions (PCRs) conducted by targeting various genes to detect *B. pseudomallei* have resulted in different limits of detection (LODs) [31,32,33]. PCR can produce false-negative results when tested on direct blood specimens due to low bacterial loads (<10 CFU/mL), which are usually below the detection limits of any PCR assay, and the presence of inhibitory substances in the blood [22].

Another diagnostic test is the InBiOS Active Melioidosis Detect (AMD), a lateral flow immunoassay (LFI) where a nitrocellulose membrane strip is coated with monoclonal antibodies (mAb 3C5) for the detection of the CPS of *B. pseudomallei* in clinical specimens [34]. It is a promising tool for the detection of *B. pseudomallei* globally and meets the criteria of being affordable, sensitive, specific, user friendly, rapid and robust, equipment-free, and delivered (ASSURED). However, it provides poor sensitivity for non-blood samples; thus, it is not yet a true point-of-care (POC) test for the diagnosis of melioidosis [25].

Various antigenic proteins have been used to develop detection methods for *B. pseudomallei*. One is the O-polysaccharide (OPS) component of lipopolysaccharide (LPS), an appealing candidate for rapid POC tests. However, variations in the OPS antigen (type A, B, B2, or rough) among *B. pseudomallei* from different geographic regions result in false negatives [35]. Another protein is hemolysin-coregulated protein 1 (Hcp1), a component of the virulence-associated type VI secretion system. The Hcp1 produced by *B. pseudomallei* is structurally different from that of *B. thailandensis*; thus, seropositivity to this antigen is less prevalent than that to OPS among healthy individuals in endemic areas [26], making the use of Hcp1 preferable to that of OPS for detecting *B. pseudomallei* antibodies. The chaperonin GroEL is a heat-shock protein considered as a promising serodiagnosis antigen for discriminating melioidosis cases from healthy individuals [28,36]. However, *Pseudomonas* spp. and other Gram-negative non-fermentatives (GNFs) frequently lead to false-positive results, suggesting that the protein shares cross-reactive epitopes among these bacteria [37]. Other alternative antigens that might be useful for the serodiagnosis of melioidosis are culture filtrate (CF) and whole-cell (WC) antigens. However, these antigens have the drawbacks of unstandardized preparation methods and are therefore not reproducible across laboratories [35].

Another potential antigenic protein should be investigated for the rapid detection of *B. pseudomallei*, especially from direct clinical specimens, in order to initiate antibiotic treatment to prevent relapse and reduce the mortality rate. Hence, for this review, we analyzed the literature about BipD, a potential target for detecting *B. pseudomallei*. We discuss the structure, functions in pathogenesis, and serodiagnostic approaches for BipD, including its detection using aptasensors as a possible promising diagnostic method.

## 2. Content

### 2.1. Type III Secretion Systems (T3SSs) of B. pseudomallei

**Figure 1:**
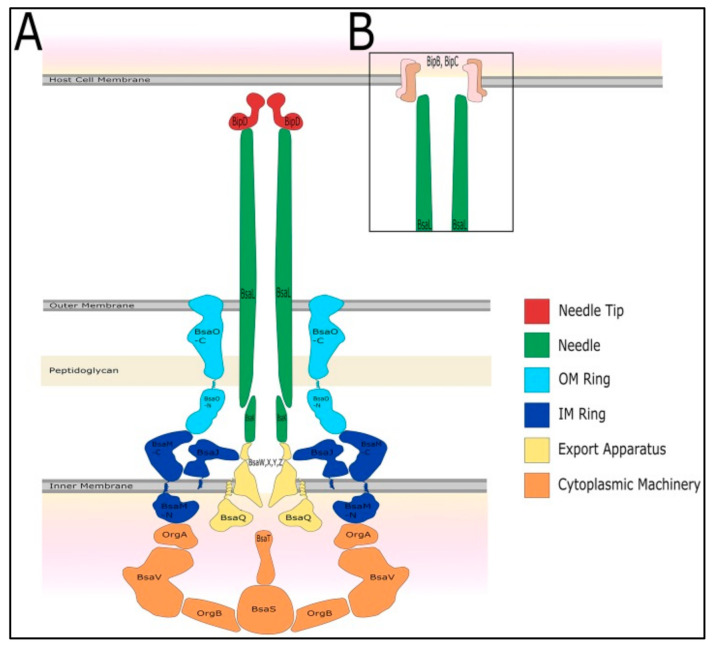
The predicted structure of the type III secretion system 3 (T3SS-3) of *B. pseudomallei*. Adapted with permission from [42]. Copyright © 2017 Vander Broek and Stevens. (**A**) The T3SS-3 comprises of cytoplasmic machinery, export apparatus, inner and outer membrane rings, needle, needle tip (*Burkholderia* invasion protein D, BipD), and (**B**) translocon proteins (*Burkholderia* invasion protein B, BipB and *Burkholderia* invasion protein C, BipC).

Type III secretion systems (T3SSs) are structurally related to bacterial flagellum systems. Many pathogenic Gram-negative bacteria use T3SSs to deliver effector proteins into host cells to facilitate their own survival and colonization [38]. Three T3SSs (T3SS-1, T3SS-2, and T3SS-3) are encoded in the *B. pseudomallei* genome on chromosome 2 [39]. T3SS-3 is the best-characterized T3SS of *B. pseudomallei*, and it is also known as *Burkholderia* secretion apparatus (Bsa). Bsa is a member of the Inv/Mxi-Spa family of T3SSs from *Shigella flexneri* and *Salmonella* spp. (SPI-1) [40]. It is often referred to as an injectisome and is composed of approximately 20 different proteins that assemble a nanosyringe [41]. Bsa comprises the structural components of the apparatus (the basal body spans both bacterial membranes, and an extracellular needle protrudes from the bacterial surface) as shown in Figure 1, secreted proteins (translocators and effectors), chaperones, and cytoplasmic regulators [42]. Similarly to its *Salmonella* and *Shigella* homologs, Bsa follows the inside-out model for its assembly [43].

### 2.2. BipD of Bsa

BipD is a dumbbell-shaped protein at the Bsa needle tip and is homologous to SipD (*Salmonella*) (26% identity; 36% similarity), IpaD (*Shigella*) (27% identity; 39% similarity), and LcrV (*Yersinia*) [42]. BipD is different from IpaD and SipD because it does not contain any cysteine residues; both IpaD and SipD possess one cysteine at different positions [44]. BipD serves as a platform for the assembly of the translocon pore with *Burkholderia* invasion protein B (BipB) (a major translocon protein) and *Burkholderia* invasion protein C (BipC) (a minor translocon protein). It allows for the direct passage of effector proteins into the target host cell from the cytoplasm of *B. pseudomallei*. The effector proteins suppress host cell processes to benefit the bacteria [42]. *Shigella* IpaD is involved in regulating the secretion of other translocator and effector proteins by interacting with the gatekeeper protein MxiC [45]. Since BipD is similar to IpaD, an interaction between BipD and BsaP is suggested to perform a similar function in *B. pseudomallei*. The tip protein complex exists in two states, as shown in *Salmonella* and *Shigella* [46,47]. The first is a closed state that prevents the secretion of the other translocon proteins (BipB and BipC) from the needle. The second is an open state that occurs when bacteria interact with the host cells. The other translocon proteins are secreted from the needle to form the translocon pores on the host cell membrane.

### 2.3. The Three-Dimensional (3D) Structure of BipD

BipD is encoded by the *bipD* gene, and it consists of 310 amino acids with a molecular mass of approximately 34 kDa. The precise structure of BipD was identified from a selenomethionyl-BipD (SeMet-BipD) crystal, obtained using the hanging-drop method, followed by diffraction at a resolution of 2.1 Å [48]. The Research Collaboratory for Structural Bioinformatics (RCSB) Protein Data Bank (PDB) allocated accession number is 2izp for the deposited coordinates and structure factors. Another structural analysis of BipD was performed in the lipid head-group phosphocholine. This BipD crystal diffracted to 1.5 Å, followed by structural refinement at a near-atomic resolution [49]. The coordinates and structure factors were deposited in the PDB with the accession number 3nft.

**Figure 2:**
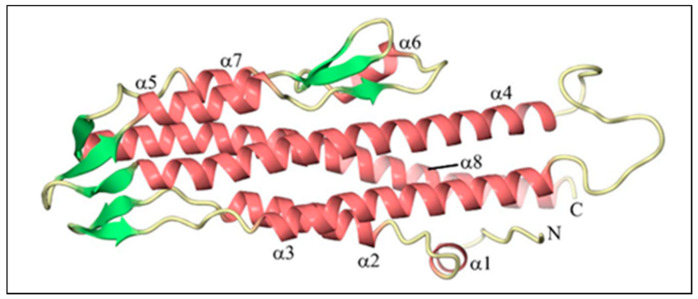
The tertiary structure of *Burkholderia* invasion protein (BipD) generated using CueMol at 2.1 Å resolution. The pink color indicates helices, whereas the green color represents strands. Adapted with permission from [44]. Copyright © 2006 Elsevier Ltd.

BipD is mainly composed of a bundle of α-helical segments in an anti-parallel configuration with two three-stranded β-sheet regions (Table 1): one of them at one end of the bundle, and the others on the opposite side, as shown in Figure 2. The crystal structure is consistent with far-UV circular dichroism (CD) spectra, confirming the marked dominance of α-helices over β-sheets in the tertiary structure [44]. BipD is also characterized by an extended helical coiled coil assembled between the helices at the middle and *C*-terminus of the sequence. Two helices (α1 and α2) from the *N*-terminal domain fold against one end of the coiled coil, while another two helices (α4 and α6) from the *C*-terminal domain pack against the other end of the coil. Therefore, BipD comprises two four-helix bundles that share a long coiled coil [50].

**Figure 3:**
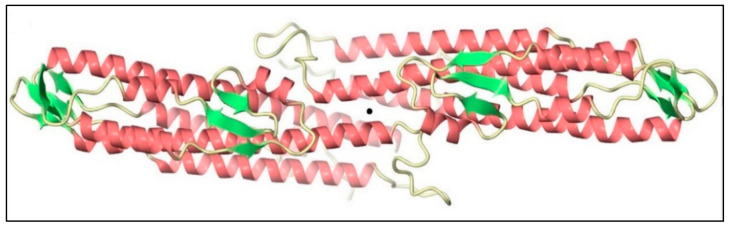
Two monomers of *Burkholderia* invasion protein D (BipD) within the crystallographic asymmetric unit generated using CueMol. The central black sphere shows that both monomers are connected by helices 4 and 8 with an approximately two-fold symmetry. Adapted with permission from [44]. Copyright © 2006 Elsevier Ltd.

Helix 8 is a remarkably conserved part of the BipD structure, and it plays a crucial role in the formation of the dimer. Figure 3 shows two BipD molecules in the crystallographic asymmetric unit that are connected by creating extensive contacts from both subunits involving the *C*-terminal end of helix 8 and the *N*-terminal end of helix 4 in an anti-parallel manner [44]. Notably, another study also showed the dimer interface, but the only difference was that the two monomers were slightly reoriented [49]. The total surface-accessible area of each monomer buried in this putative dimer interface (1271 Å^2^) is larger than that for the previous crystal form (970 Å^2^) [44].

No specific chaperones have been identified for BipD to date. Chaperones should help the movement of proteins through the needle by partially or entirely unfolding them. The *N*-terminal domain of BipD chaperones residues on the coiled coil, which is involved in interactions with the needle [50]. The elongated structure of BipD permits it to pass through the needle pore with minimal unfolding. The region formed by residues 171–250 may unfold from the four-helix bundle to reduce the molecule’s diameter to approximately 25 Å, which matches the needle’s inner diameter [44].

### 2.4. Functions of BipD in the Pathogenesis of B. pseudomallei

*B. pseudomallei* is an opportunistic and intracellular facultative pathogen, whose pathogenesis depends on intrinsic virulence factors, including Bsa [39]. It is capable of extracellular growth and survival because it can resist complement-mediated killing in the human serum. However, the intracellular growth of *B. pseudomallei* is essential for its virulence [51].

**Figure 4:**
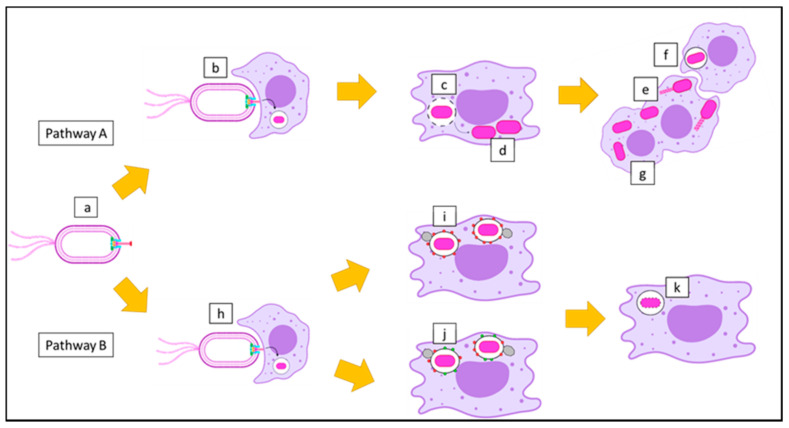
Postulation of the intracellular survival of *B. pseudomallei*. (**a**) A motile Gram-negative *B. pseudomallei* with *Burkholderia* secretion apparatus (Bsa) can use two pathways after infection of host cells. (**b**) Invasion of *B. pseudomallei* into a macrophage, where they are internalized into the phagosome. (**c**) *B. pseudomallei* escapes from the phagosome by rupturing the phagosome membrane, and (**d**) replicates in the cytoplasm of the macrophage. (**e**) *B. pseudomallei* forms actin tails, which leads to membrane protrusion that allows (**f**) cell-to-cell spread, and ultimately results in (**g**) the formation of multinucleated giant cells (MNGCs) (pathway A). On the other hand, (**h**) *B. pseudomallei* can be phagocytosed by a macrophage and trapped in the phagosome. (**i**) Bacterium-containing phagosomes undergo phagosome maturation with lysosomal-associated membrane protein 1 (LAMP-1). (**j**) In some cases, bacterium-containing phagosomes undergo LC3-associated phagocytosis (LAP) processes, where phagosome maturation occurs with both LAMP-1 and microtubule-associated protein 1A/1B-light chain 3 (LC3). (**k**) Finally, the macrophage destroys *B. pseudomallei* with the assistance of the phagolysosome (pathway B).

*B. pseudomallei* invades phagocytic cells such as polymorphonuclear leukocytes and macrophages via phagocytosis. With the help of functional Bsa, it also enters non-phagocytic cells by inducing its own uptake. *B. pseudomallei* escapes from the phagosome by lysing the phagosome membrane as early as 15 min after internalization, and replicates in the host cytoplasm [52]. *B. pseudomallei* exploits the host cell cytoskeleton by inducing actin polymerization at one pole of the bacterium to produce actin tails that propel it throughout the host cytoplasm. It also forms membrane protrusions into adjacent cells to promote cell-to-cell spread. On contact with neighboring cells, it causes cell fusion and the development of multinucleated giant cells (MNGCs) that contain hundreds of nuclei (pathway A), as shown in Figure 4 [53,54].

Some *B. pseudomallei* cannot escape from the phagosome due to defects in the Bsa, including the mutation of BipD. Microtubule-associated protein 1A/1B-light chain 3 (LC3), is recruited to bacterium-containing phagosomes to stimulate LC3-associated phagocytosis (LAP). LC3 promotes phagosomal maturation through the recruitment of other proteins, including lysosomal-associated membrane protein 1 (LAMP-1). Both proteins induce the fusion of the phagosome with the lysosome, which leads to bacterial killing. The phagosome also matures into a phagolysosome with the recruitment of LAMP-1 only (Figure 4, pathway B) [55].

Several studies have been performed to assess the role of BipD in the development of melioidosis. A *bipD* mutant was constructed to reveal its contribution to pathogenesis, as summarized in Table 2. Firstly, no variations were detected in the in vitro growth and secreted protein profiles between the wild-type strain and the *bipD* mutant in lysogeny broth (LB) at 37 °C [40,56]. Both studies indicated that BipD does not affect the in vitro growth rate of *B. pseudomallei* in culture broth. Secondly, the invasion of wild-type *B. pseudomallei* and the *bipD* mutant into cultured HeLa cells occurred at a low frequency compared to that of a control *Salmonella typhimurium* strain [40]. However, another study showed that the *bipD* mutant caused a significant reduction in the invasion of HeLa cells and suggested that other Bsa-secreted proteins may be involved in *B. pseudomallei* uptake by non-phagocytic cells [57].

The *bipD* mutant showed a marked decrease in intracellular replication within J774.2 murine macrophage-like cells due to the confinement of the *bipD* mutant to the endosome [40]. The spleens and livers of mice infected with the wild-type strain were swollen and contained many abscesses, whereas those infected with the *bipD* mutant developed less-prominent abscesses and splenomegaly [56]. These findings demonstrate that BipD has a vital role in promoting the intracellular replication of *B. pseudomallei*. Furthermore, J774.2 murine macrophage-like cells infected with the wild-type strain showed peripheral membrane protrusions with extreme filamentous actin staining at one pole of the intracellular bacterium [53]; by contrast, no such protrusions or actin rearrangement was detected in any of the cells infected with the *bipD* mutant, even when the bacterial load was very high [55,56]. These observations suggest that BipD can induce membrane protrusions by allowing *B. pseudomallei* to access cytoplasmic actin.

The *bipD* mutant also displayed almost no escape from the phagosome at 2 and 4 h after the infection of RAW 264.7 macrophage cells. However, a small number of free *bipD* mutants were found at 6 h post-infection, indicating that the *bipD* mutant could escape the phagosome later [55]. Therefore, BipD is not the only protein that helps *B. pseudomallei* to escape from the phagosome into the cytoplasm. The *bipD* mutant also exhibited lower intracellular survival at 6 h after the infection of the RAW 264.7 macrophage cells due to the LAP process and the recruitment of LAMP-1 [55]. Ninety-six percent of the LAMP-1-associated *bipD* mutants were identified in the J774.2 murine macrophage-like cells, suggesting that they were unable to escape from the phagosome, which led to the fusion of lysosomes with bacterium-containing vacuoles [57]. Thus, BipD promotes the intracellular survival of *B. pseudomallei* by allowing them to escape from phagosomes.

Hence, we conclude that BipD is a protein that can be categorized as a virulence factor because of its ability to enable the bacterium to invade non-phagocytic cells, escape from the phagosome, and induce intracellular replication. It also forms actin tails, causing cell-to-cell spread, thus allowing the bacterium to survive the extracellular environment and evade both host defenses and antibiotic treatment [58].

### 2.5. Detection of BipD-Specific Antibodies for Diagnosis

#### Serological Tests

A rapid, cost-effective, and sensitive serological assay is required to diagnose melioidosis. The use of crude antigens leads to poor specificity due to cross-reactions with antibodies against other bacteria [59]. Therefore, the use of recombinant antigens has been considered in the serodiagnosis of melioidosis. Druar et al. demonstrated a high degree of BipD identity in the other *B. pseudomallei* strains [60]. The amino acid residues of BipD displayed limited or no identity to other proteins in *Mycobacterium tuberculosis*, the causative agent of tuberculosis and the disease that melioidosis mimics. These findings highlighted the potential for the use of BipD as a target in diagnostic tests for melioidosis [61].

Two studies were performed using indirect ELISA to detect specific antibodies against recombinant BipD (rBipD). The first study identified BipD-specific antibodies in the sera of culture-confirmed melioidosis patients from Malaysia, Thailand, and Australia. The BipD ELISA demonstrated moderate sensitivity (42%) but high specificity (100%). This study also showed that no detectable antibody responses were found in healthy individuals from the endemic region (Thailand) when recombinant BipD was used compared to crude antigens in IHA [61]. The other study was conducted to determine BipD-specific antibodies in patient sera from Thailand and Australia [60]. A statistical evaluation showed that ELISA had worse diagnostic accuracy than the existing IHA for the Thai serum samples: the sensitivity and specificity were 63% and 61% for the BipD ELISA, whereas they were 72% and 62% for the IHA, respectively. Similar results were shown in a statistical examination of Australian serum samples. The BipD ELISA had lower diagnostic sensitivity (75% vs. 76%) and specificity (64% vs. 99%) than the IHA that was previously conducted at Townsville Hospital [62]. There are also several limitations of ELISA such as its requirement for serum dilution steps and a microtiter plate reader, and the optical density (OD) cut-off values may need to be determined for different areas based on the levels of exposure of local populations to *B. pseudomallei* or antigenically related organisms [35]. The determination of the cut-off value is vital for the interpretation of the results. Cut-off values too low or high may lead to false-negative or false-positive results [63].

The immunoblotting technique was also used to analyze BipD-specific antibodies in serum samples from Thailand [64]. An elevation in BipD sensitivity (from 78% to 100%) was obtained after the removal of the glutathione S-transferases (GST), because the presence of GST prevents the binding of BipD to antibodies. However, the specificity of BipD (91.1%) was not statistically different from that of fusion GST–BipD (90%) due to the cross-reactivity of BipD and high background during melioidosis infection. Stevens et al. found that BipD-specific antibodies could be detected using the immunoblotting method, as the serum from a convalescent melioidosis patient reacted with GST–BipD. No reactivity was observed with normal human serum [40].

Surface plasmon resonance (SPR) biosensors have become an extremely potent tool due to their high sensitivity and real-time monitoring capacity [65]. BipD antibodies were detected by SPR using serum samples from Thailand, which resulted in 100% sensitivity and specificity [66]. SPR has several advantages in serodiagnosis, such as only a small amount of serum being required, the results being available in only 20 min (allowing infected patients to initiate proper care or therapy much earlier), and the fact that one electrode preparation can be used up to 30 times (which saves time and reduces the analysis cost).

BipD-specific antibodies were detected with varying sensitivities and specificities based on the method performed, as shown in Table 3. The disadvantages of the serodiagnosis of BipD are the cross-reaction of BipD with other antibodies, the high seropositivity among the healthy population, and the fact that not all patients infected with *B. pseudomallei* produce sufficient levels of antibodies against BipD [64,66]. The main drawback of all the serological assays is that the antibodies can only be detected 7–14 days post-infection [33]. Thus, antibody-detection methods cause delays in both the diagnosis and treatment of melioidosis. Therefore, BipD can be used in antigen-detection methods for the rapid diagnosis of melioidosis.

## 3. Conclusions

Melioidosis is a severe bacterial infection caused by *B. pseudomallei.* Rapid antimicrobial therapy is needed to improve patient outcomes, which highlights the need for antigen detection for the rapid determination of *B. pseudomallei* in clinical samples. Hence, we reviewed information about BipD, a needle tip protein secreted by Bsa. As outlined in this review, BipD consists of abundant α-helices and some β-strands, and it exists as a dimer under biological conditions. It has principal roles in pathogenesis, such as facilitating the invasion of non-phagocytic cells, escape from the phagosome, the induction of intracellular replication, and the formation of actin tails. Several attempts have been made to detect BipD-specific antibodies from human sera using ELISA, immunoblotting, and SPR; however, none have been accepted for detecting *B. pseudomallei* in clinical samples. Therefore, we suggest that BipD be used in antigen-detection assays to directly indicate the presence of *B. pseudomallei.*

## 4. Future Directions

Aptamers are short (generally 15–80 nucleotides), single-stranded oligonucleotides (ssDNA or ssRNA) that were discovered in 1990 [67]. The name aptamer comes from the Greek “aptus” and “merus”, meaning “to fit” and “particle”, respectively. Aptamers are also called chemical antibodies [68]. They are selected against a target of interest from synthetic ssDNA or ssRNA libraries, generally through a process termed the Systematic Evolution of Ligands by EXponential enrichment (SELEX) [69].

Aptamers have some essential benefits that make them perfect candidates for supplanting antibodies. They can fold into stable 3D structures that allow them to interact with a wide range of targets, such as small molecules (ions), peptides, proteins, and cells. The dissociation constants (Kd) of aptamers toward their targets are in the micro- to picomolar range [70]. They are more resistant to pH and temperature changes and able to withstand long-term shelf storage at room temperature while retaining their unique tertiary structures and activities [71]. Once selected, aptamers can undergo subsequent amplification through PCR to produce high-purity aptamers in large quantities with minimal batch-to-batch variation [69].

The simple chemical structure of an aptamer makes it amenable to further modifications with functional groups according to specific purposes. Their production does not require any animals or specific cell lines; thus, they can be raised against toxic molecules or non-immunogenic targets with high affinity and specificity [72]. Aptamers are promising affinity ligands for several appealing applications (such as basic science, chemical sensing, clinical diagnosis, targeted drug delivery, and therapy) due to their low molecular weights, convenient chemical synthesis and alteration, and rapid tissue penetration [73].

An aptasensor is a biosensor in which an aptamer is used as a biological recognition element and is classified as electrochemical, optical, or mass sensitive according to the signal harvesting method. Aptasensors have attracted particular attention in recent years due to their small sizes. Their versatility allows them to be immobilized efficiently at a high density; these criteria are essential for the development of miniaturized multiplexing systems [74]. POC tests and clinical treatments require the measurement of diagnostically relevant protein biomarkers. Aptamers recognize proteins more quickly than small molecules or ions [75]. Identifying the presence or absence of pathogens or species-specific proteins is vital in clinical settings. It can also be applied as a direct intervention in cases when early diagnosis and management are required. Aptasensors are a promising novel approach for the sensitive and rapid diagnosis of clinical pathogens [76]. Various detection methods can be used with aptasensors for the identification of pathogens, including colorimetry, fluorescence, chemiluminescence, LFA, electrochemical impedance spectroscopy (EIS), and surface plasmon resonance (SPR) [77,78].

Hence, an aptamer targeting BipD appears to be a potential diagnostic approach for the identification of *B. pseudomallei* from direct clinical samples. Gnanam et al. aimed to create an RNA aptamer via the SELEX technique for three recombinant proteins of *B. pseudomallei*, including BipD [79]. Highly pure forms of all three proteins were obtained using affinity chromatography. However, the in vitro selection of aptamers only proceeded until the third round, so no final aptamer sequences were obtained from this analysis.

Therefore, for future detection methods, we propose the development of a DNA aptamer against BipD, as DNA aptamers have greater advantages compared with RNA aptamers. DNA aptamers are more stable (including in human serum), do not require additional steps such as reverse transcription, and are cost-effective to produce [80,81]. In addition, we also recommend the use of bioinformatics tools and computational methods in aptamer screening and aptamer–target complex identification to overcome the disadvantages of SELEX techniques, which are tedious, time-consuming (usually taking several weeks to complete), and not cost-efficient [82].

## Figures and Tables

**Table 1:** Residues of *Burkholderia* invasion protein D (BipD) that represent structural domains. Adapted from [44].

Residues of BipD	Structural Domains
36–43	α-helix (helix 1)
47–63	α-helix (helix 2)
64–81	β-hairpin (β1 and β2)
82–111	α-helix (helix 3) runs anti-parallel to helix 2
128–170	α-helix (helix 4) runs anti-parallel to helix 3
171–183	β-hairpin (β3 and β4)
184–196	α-helix (helix 5) runs anti-parallel to helix 4
197–203	β-hairpin (β5), last three residues are part of a three-stranded β-sheet with β6 and β7
209–216	α-helix (helix 6)
220–230	β-hairpin (β6 and β7)
233–241	α-helix (helix 7) runs anti-parallel to helix 5
246–250	β-hairpin (β8) forms a three-stranded β-sheet with β3 and β4
251–301	α-helix (helix 8)

**Table 2:** Characterization of *bipD* mutants in the pathogenesis of melioidosis.

Wild-Type Strain of *BipD* Mutant	In Vitro Growth Rate in LB at 37 °C	Escape from the Phagosomes of Macrophage Cells	Intracellular Replication	Invasion of Cultured HeLa Cells	Induced Membrane Protrusions and Actin Tails in Macrophage Cells	Association of Intracellular Bacteria with LAMP-1-Containing Vacuoles in Macrophage Cells	Refs.
10276	No effect	ND	Marked reduction in intracellular replication	The invasion occurred at a low frequency	No protrusions or actin rearrangements	Increased association with LAMP-1-containing vacuoles	[40]
10276	ND ^a^	ND	ND	Highly significant reduction in invasion	ND	ND	[57]
576	No effect	ND	Low replication of the bacteria in the liver and spleen	ND	ND	ND	[56]
K96243	ND	Unable to escape from phagosomes and showed a high level of co-localization with LC3	ND	ND	No formation of actin tails	Increased levels of co-localization with LAMP-1	[55]

^a^ ND: not determined; LB, lysogeny broth; LAMP-1, lysosomal-associated membrane protein 1.

**Table 3:** Serodiagnostic assays using *Burkholderia* invasion protein (BipD) as the target.

Methods	BipD	Population	Sensitivity (%)	Specificity (%)	Refs.
ELISA	Histidine-BipD (His–BipD)	Malaysia, Thailand, and Australia	42	100	[61]
ELISA	His–BipD	Thailand and Australia	63–75	61–64	[60]
Immunoblot	Glutathione S-transferases-BipD (GST–BipD) BipD	Thailand	78 100	90 91.1	[64]
Immunoblot	GST–BipD	ND ^a^	ND	ND	[40]
Surface plasmon resonance (SPR)	BipD	Thailand	100	100	[66]

^a^ ND: not determined.

## Data Availability

Data have been taken from previous studies as cited in Table 1, Table 2 and Table 3.
